# Reform of the Medical Acts

**Published:** 1898-01-08

**Authors:** 


### Reform of the Medical Acts.

It is becoming abundantly clear that some far more stringent control over medical practice than exists at present must be obtained if the public, and especially if the poorer classes, are to be protected from the evils and the dangers which arise from the growing pretensions of unqualified practitioners. We do not think that the Legislature will ever go to the length of making it illegal for people to give advice to each other in regard to the treatment of their diseases. To ask such a thing would be absurd. False pretence, however, is another matter, and one which ought to be provided for by law. It is just as necessary now as it was at the time of the passing of the Medical Act of 1858 that persons requiring medical aid should be able to distinguish between qualified and unqualified medical men. To enable them to do so was the object with which the Act was passed, yet it is obvious to all men that as the law is at present administered neither the profession nor the public is protected.

In coming to any decision as to the nature of the reforms in the Medical Acts which are necessary much must depend upon the answer to the question—How far would the present Acts carry us if properly enforced? From a paper recently read by Professor Horsley he appears to be of the opinion that the Act of 1858 already provides that we, "being registered practitioners of medicine, are alone qualified practitioners of medicine," that a person not registered is not entitled to practise, and that if he does so he should be punished.

We do not, however, think that the words bear the interpretation placed upon them by Professor Horsley. What Section 40 does say is that "any person who shall wilfully and falsely take or use the name or title of a physician, doctor of medicine, licentiate in medicine and surgery, bachelor of medicine, surgeon, general practitioner, or apothecary, or any name, title, addition, or description implying that he is registered under this Act, or that he is recognised by law as a physician, &c., shall upon a summary conviction for any such offence pay a sum not exceeding £20." There is nothing here about unregistered persons not being entitled to practise, nor about their being punished for so doing. The whole question depends upon what name, title, addition, or description can be taken to imply that a man is registered, and it is here that difficulties have arisen. What we really want is some modification of this clause making it embrace, as well as the assumption of a title, the doing of anything which shall lead people to believe that a person is a qualified medical practitioner. It is the open assumption of the position of the medical practitioner by people who are unqualified that deludes the public and ruins the profession.

If unqualified people could be debarred from seeing "patients," having "consulting rooms," going their "rounds," and otherwise posing as medical men, a little counter practice by chemists and a little quacking by old women would be a small affair. We do not think there is any deception about counter practice. The people know quite well that it is a chemist that they are getting their physic from; and, indeed, unless the medical profession is prepared to sell a powder for a penny over the counter, we do not see what is to be the substitute for counter practice as it is conducted in some districts, and, as to the old women, there is certainly no deception there.

When, however, the chemist has a consulting room behind his shop, and "goes his rounds" for all the world "just like a doctor," and when the unqualified man puts his name on one door as "Mr. So-and-So," and on another puts a plate inscribed "Surgery," and also goes his "rounds," and sees his "patients" and does his "operations," and when a man advertises that he is a specialist for certain diseases, and sees "patients," at certain hours at his "consulting rooms," even though these people take no name and use no title implying that they are registered, they cheat the public and make people think that they are qualified. In this way the very intention of the Act is evaded, and its provisions are rendered of no avail. This is the first and the great point which requires alteration. No doubt it is a difficult thing to draft any set of words that shall cover such slippery fellows as we have here to deal with, but the aims and objects of the old Act are plain, and it is equally plain that from defective wording it has failed to have the effect that was intended by its framers. If Professor Horsley can do better still, by all means let us support him in all he may propose; but this, at least, we can properly ask from any Parliament, and from any party, without the slightest stigma of self-seeking, viz., that the Medical Acts shall be so modified as to put a stop to a peculiarly dangerous form of fraud, which is undoubtedly the cause of much loss of life. The Medical Acts were intended to put a stop to false pretence; let them be made to do so.

